# Malaria diagnostic and treatment practices for febrile children under 5 years at two general hospitals in Karamoja, a high transmission setting in Uganda

**DOI:** 10.1186/s12936-022-04329-w

**Published:** 2022-11-03

**Authors:** Jane Frances Zalwango, Joaniter I. Nankabirwa, Freddy Eric Kitutu, Rebecca Akunzirwe, Remmy Buhuguru, Joan Bayowa Rokani, Emmanuel Ssendikwanawa, Sarah Kiguli, Emmanuel Arinaitwe, Joan N. Kalyango

**Affiliations:** 1 Clinical Epidemiology Unit, School of Medicine, Makerere University College of Health Sciences, Kampala, Uganda; 2 Infectious Diseases Research Collaboration, Kampala, Uganda; 3 Department of Pharmacy, School of Health Sciences, Makerere University, Kampala, Uganda; 4 Department of Pediatrics and Child Health, School of Medicine, Makerere University College of Health Sciences, Kampala, Uganda; 5 Sustainable Pharmaceutical Systems (SPS) Unit, School of Health Sciences, Makerere University, Kampala, Uganda

**Keywords:** Malaria case management, Prevalence, Inappropriate case management, Factors associated

## Abstract

**Background:**

Malaria is one of the leading causes of morbidity and mortality among children under 5 years of age in Uganda. Although Karamoja sub-region has the highest prevalence of malaria, and one of the highest case fatality rates in children under 5 years, information on malaria case management for the sub-region is scarce. The study evaluated the malaria diagnostic and treatment practices, as well as the factors associated with inappropriate care for children under 5 years of age presenting with fever in two public hospitals within the sub-region.

**Methods:**

A cross-sectional study was conducted amongst 857 children under 5 years of age who presented with fever at Abim and Kaabong general hospitals between February and March 2020. A questionnaire was administered to the primary caregiver during exit/bedside interviews to collect socio-demographic information. The participant clinical notes were reviewed to capture information on laboratory tests conducted, diagnosis given, and treatment prescribed. In addition, a health facility assessment was conducted and information on healthcare workers was collected. The healthcare worker and facility data was linked to the participant’s hospital visit. Main outcome measures were malaria diagnostic and treatment practices.

**Results:**

Of the 857 children enrolled, 820 (95.7%) had a malaria diagnostic test done and 623 (76.0%) tested positive for malaria. All test positive children received anti-malarial treatment, however, only 424/623 (68.1%) received the recommended anti-malarial drug and 376/424 (88.7%) received the right dose of the treatment. Inappropriate diagnosis/treatment was in 321 (37.5%) of the enrolled participants. Factors associated with inappropriate diagnosis/treatment included: lack of recommended anti-malarials on the day of the visit (Prevalence Ratio [PR] = 2.1, 95% confidence interval [CI] 1.8–2.4), hospital where care was sought (PR = 0.4, 95% CI 0.3–0.5), being managed by a recently supervised health worker (PR = 0.5, 95% CI 0.2–0.9), and health worker cadre (PR = 0.8, 95% CI 0.7–0.9).

**Conclusion:**

The prevalence of inappropriate malaria diagnosis and treatment in the Karamoja sub-region was high with approximately one in every three children receiving inappropriate care. This was majorly influenced by health system factors, which if improved upon may reduce malaria-related mortalities in the sub-region a vital step in meeting the country’s target of zero deaths from malaria by 2030.

## Background

Despite the roll out of effective control interventions, malaria remains one of the most important global health challenges, with the highest burden occurring in the World Health Organization (WHO) African region. In 2020, the WHO African Region, accounted for 95% of all global malaria cases at an estimated 228 million cases [1]. Uganda, in third place at 5% contribution, was one of six countries that accounted for 55% of all malaria cases [1]. Malaria is the leading cause of morbidity and mortality in the country, accounting for 29.1% of outpatient visits, 39.5% of inpatient admissions, and 10.9% of hospital deaths [2]. The bulk of the disease is in children under 5 years.

With such a heavy burden, appropriate case management (prompt and effective malaria diagnosis and treatment) with a primary objective of ensuring rapid and complete elimination of *Plasmodium* parasites is critical to reduce transmission, prevent disease progression to severe states, complications, and malaria-related mortality [3]. However, if case management is compromised combined with existing health system challenges of inadequate health care resources, lack of or ineffective use of control measures, limited access to care, delays in diagnosis and treatment initiation, and delays in seeking care; malaria-related mortalities are inevitable [4, 5]. To improve patient outcomes, Uganda adopted the test and treat policy in 2016 [6] in response to the WHO recommendation that all suspected malaria cases undergo a parasitological test (microscopy or RDT) to confirm the diagnosis enabling health workers quickly distinguish between malarial and non-malarial fevers facilitating appropriate treatment, and those who test positive for malaria be treated within 24 h [3]. This prompt and effective diagnosis and treatment of cases combined with continued implementation of other malaria control interventions such as long-lasting insecticidal nets (LLINs) and indoor residual spraying (IRS) has contributed substantially to the decline in malaria morbidity and mortality in the country. Data from the 2018/2019 malaria indicator survey shows a 30% decline in the markers of infection and disease compared to estimates from 2014 [7, 8]. Unfortunately, these declines have not been uniform across the country [8].

The Karamoja sub-region is one of the high malaria burden areas in Uganda and its malaria parasite prevalence estimates by microscopy are at 34% amongst children under 5 years which is almost four times above the national average of 9% [8]. The sub-region also has one of the highest reported malaria mortality rates in the country [9]. Unfortunately, the specific contributors to this high mortality among children living in Karamoja region has not been well documented. This study evaluated one of the known risk factors for malaria-related mortality; case management (focusing on malaria diagnostic and treatment practices) of children under 5 years presenting with fever at the two public hospitals in the Karamoja sub-region. The study also assessed the factors associated with inappropriate practices in this population.

## Methods

### Study setting

The study was conducted in Abim and Kaabong general hospitals. The two hospitals are part of the four government-owned general hospitals in the Karamoja sub-region. Both hospitals provide preventive, in-patient, and outpatient medical services free of charge [10]. The Karamoja sub-region is one of the highest malaria transmission areas in Uganda with its parasite prevalence amongst children under 5 years estimated at 34% [8]. Malaria transmission in the sub-region is highest during the long rainy period from March to September [11]. Malaria control interventions in the region have focused on the use of LLINs, case management, and intermittent preventive treatment in pregnancy. Abim and Kaabong general hospitals reported the highest burden of malaria in children under-5 years in the sub-region in 2019 as recorded in the District Health Information Software 2 (DHIS 2). The malaria related mortality was estimated at 14.8/100,000 population in Abim and 32.7/100,000 population in Kaabong in the same period [9].

### Study design, participant enrollment, and data collection

A cross-sectional study to determine the malaria case management practices and associated factors in children under-five was conducted at the outpatient and inpatient departments of Abim and Kaabong general hospitals between February and March 2020. Patients presenting to the outpatient’s departments and inpatient discharge points during the study period were consecutively screened for eligibility to participate in the study and exit interviews and chart reviews were conducted for all enrolled participants. Participants were enrolled in the study if they fulfilled the following criteria: (1) aged under 5 years; (2) had a documented or reported fever (history of fever the past 48 h or axillary temperature ≥ 37.5 °C), (3) parent or guardian has provided written consent to participate in the study. Every morning, the first participants were chosen at random from among the first seven in each department. Interviews were conducted on all enrolled participants using a detailed structured questionnaire. The questionnaire captured information on the care-givers’ or participants’ social-demographic characteristics, use of malaria preventive measures, and malaria treatment history. Information on presenting complaints, routine diagnostic procedures performed, laboratory results, and treatment prescribed were recorded from the patient-held records. A health facility checklist was filled on all days of survey to assess the presence of diagnostics and recommended anti-malarials. A healthcare worker assessment was conducted on all clinicians managing patients on the days of the survey. Health facility and health worker data were linked with participant data at analysis.

### Sample size and power calculations

Sample size was estimated using the Kish Leslie formula. Estimating that 50.6% would adhere to national malaria treatment guidelines in management of malaria [12], and using 5% sampling error and 95% level of confidence, 857 participants would be required to answer the study objectives after adjusting for a 10% non-response rate and design effect of 2.

### Data management and analysis

All data was double entered by two independent data entrants into EPIDATA version 4.4 with in-built checks to minimize errors. Data was exported into STATA Version 14 (College Station, TX: Stata Corp LP) for analysis. Baseline characteristics of the study population were summarized as percentages. Appropriate malaria care (diagnosis and treatment) was defined based on the national guidelines as: (a) having a malaria test recommended for all fever cases, (b) anti-malarial treatment only prescribed to test positive cases, (c) appropriate anti-malarial prescribed based on the national guidelines, (d) correct dose of the anti-malarial is prescribed. The primary outcome was inappropriate malaria care, which was defined as any diversion from the criterion of appropriate malaria care defined above. The proportion of inappropriate malaria diagnosis and treatment was calculated as the number of participants fulfilling the definition of inappropriate malaria diagnosis and treatment, divided by total number of participants.

Generalized linear mixed model (family-Poisson, link-log) was used to assess for the factors associated with inappropriate malaria diagnosis and treatment and reported as prevalence ratios (PR) with their 95% confidence intervals (CI). Clustering was adjusted for at facility and clinician levels while reporting robust standard errors. All variables with a p-value ≤ 0.2 at bivariate analysis or had been consistently reported in literature to be associated with malaria management practices or known confounders of clinical importance like healthcare worker training and child age were considered for multivariate analysis. Interaction and confounding were assessed and included in the final model and a p-value of 0.05 was considered statistically significant.

## Results

### Characteristics of the study population

Between February and March 2020, 857 children were enrolled into the study (no child was excluded at screening). The mean age at enrolment was 23.8 months, and 50.3% were female. Majority of the participants reported to have had fever for more than 24 h prior to seeking care (79.9%), although only 27.2% reported taking an anti-malarial prior to presentation. A total of 18 clinicians were involved in the care of the 857 participants enrolled. The mean age (SD) of the managing clinicians was 41 (9.5) years, majority were clinical officers (72.2%), and many reported not having received support supervision in the last 6 months (83.3%). Details of the participant, care-seeker, and clinician characteristics are presented in Table 1.

**Table 1:** Characteristics of the study population

Characteristic	Kaabong n (%)	Abim n (%)	Total n (%)
*Participant characteristics*			
Number	428	429	857
Male sex	212 (49.5)	214 (49.9)	426 (49.7)
Age group			
< 6 months	36 (8.4)	30 (7.0)	66 (7.7)
≥ 6 months	392 (91. 6)	399 (93.0	791 (92.3)
Primary caregiver			
Mother	413 (96.5)	411 (95.8)	824 (96.2)
Other	15 (3.5)	18 (4.2)	33 (3.9)
Educational level of caregiver			
Primary/None	395 (92.3)	303 (70. 6)	698 (81.4)
Secondary/Tertiary	33 (7.7)	126 (29.4)	159 (18. 6)
Employment status of caregiver			
None	237 (55.4)	151 (35.2)	388 (45.3)
Informal employment	191 (44. 6)	238 (55.5)	429 (50.0)
Formal employment	0 (0.0)	40 (9.3)	40 (4.7)
Time to seeking care for fever			
≤ 1 day	332 (77.7)	350 (81. 6)	682 (79.7)
> 1 day	96 (22.3)	79 (18.4)	175 (20.3)
Taken anti-malarials prior to presentation	97 (22.7)	136 (31.7)	233 (27.2)
*Health worker characteristics*			
Number	8	10	18
Mean age in years (SD)	36 (± 3.9)	45.7 (± 10.5)	41.4 (± 9.5)
Sex			
Male	8 (100.0)	9 (90.0)	17 (94.4)
Cadre			
Doctor	2 (25.0)	1 (10.0)	3 (16.7)
Clinical officer	4 (50.0)	9 (90.0)	13 (72.2)
Nurse	2 (25.0)	0 (0.0)	2 (11.1)
Training on malaria case management in last 1 year			
Yes	3 (37.5)	5 (50.0)	8 (44.4)
Supervision in last 6 months			
Yes	1 (12.5)	2 (20.0)	3 (16.7)
*Health facility characteristics*			
Days diagnostics are available			
RDTs	22 (55.0)	40 (100.0)	31 (77.5)
Microscopy	39 (97.5)	40 (100.0)	39.5 (98.8)
No test available	1 (2.5)	0 (0.0)	1 (2.5)
Days recommended anti-malarial is available			
Artemether Lumefantrine	40 (100.0)	40 (100.0)	40 (100.0)
Artesunate	22 (55.0)	40 (100.0)	31 (77.5)

### Malaria case management practices

Of the 857 children enrolled, 820 (95.7%) had a malaria test recommended and 36/37 (97.3%) without a test recommended received an anti-malarial. Of the 820 participants where a malaria test recommended, 242 (29.5%) were tested by microscopy and 578(70.5%) by RDT and 623 (76.0%) had positive test results by either microscopy or RDT. All participants with test positive results received anti-malarial treatment. However, only 424 (68.1%) received the recommended anti-malarial drug, and of these, only 376 (88.7%) were recommended the right dose of the drug (artemether/lumefantrine for uncomplicated malaria and IV artesunate for severe malaria). In the 48 participants with the wrong dozes prescribed, 32 (66.7%) received a higher dose than recommended (Table 2).

**Table 2:** Malaria case management practices for 857 children under 5 years enrolled in the study

Malaria case management practices	Kaabong, n (%) N = 428	Abim, n (%) N = 429	Overall, n (%) N = 857
Had fever and was tested			
Yes	396 (92.5)	424 (98.8)	820 (95.7)
No (Inappropriate)	**32 (7.5)**	**5 (1.2)**	**37 (4.3)**
Test results of tested participants			
Positive	331(83.6)	292(68.9)	623(76.0)
Negative	65(16.4)	132(31.1)	197(24.0)
Tested negative and prescribed an anti-malarial			
Yes (Inappropriate)	**28 (43.1)**	**9 (6.8)**	**37 (18.8)**
No	37(56.9)	123(93.2)	160(81.2)
Tested positive and prescribed an anti-malarial			
Yes	331(100.0)	292(100.0)	623(100.0)
No (Inappropriate)	**0 (0.0)**	**0 (0.0)**	**0 (0.0)**
Positive test and recommended anti-malarial prescribed			
Yes	157(47.4)	267(91.4)	424(68.1)
No (Inappropriate)	**174 (52.6)**	**25 (8.6)**	**199 (31.9)**
Correct dose of recommended anti-malarial drug prescribed			
Yes	143(91.1)	233(87.3)	376(88.7)
No (Inappropriate)	**14 (8.9)**	**34 (12.7)**	**48 (11.3)**
Overall classification			
Number enrolled	428	429	857
Appropriately managed	179 (41.8)	357 (83.2)	536 (62.5)
Inappropriately managed	249 (58.2)	72 (16.8)	321 (37.5)

**Fig. 1:**
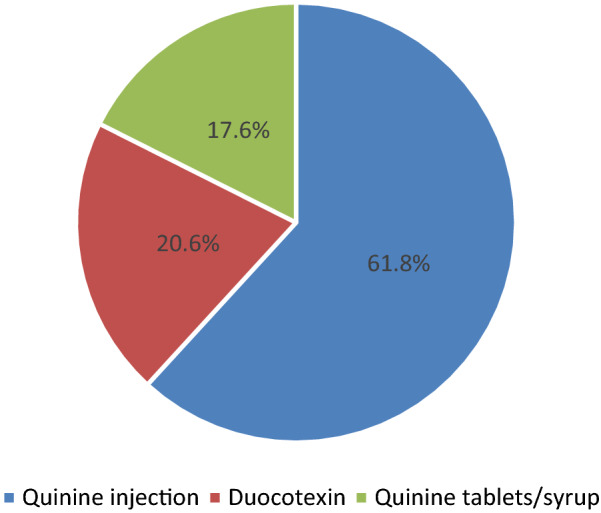
Anti-malarials other than the recommended first-line anti-malarials prescribed to children with laboratory confirmed malaria

From this analysis, 321(37.5%) participants received inappropriate malaria diagnosis and treatment (Table 2) with the largest contributor being prescription of anti-malarials other than the recommended first line anti-malarial drugs (199/321) (Fig. 1).

The commonest prescribed anti-malarial drugs other than the recommended first line treatment included (1) quinine injection (123/199, 61.8%), (2) dihydroartemisinin/piperaquine tablets (Duocotexin®) (41/199, 20.6%), and (3) quinine tablets/syrup (35/199, 17.6%) for treatment of uncomplicated malaria.

### Factors associated with inappropriate malaria care

Factors significantly associated with inappropriate malaria care at multivariate analysis included the hospital where one sought healthcare, availability of the recommended anti-malarial drug on the day that care was sought, and being managed by a cadre who had received support supervision in the last 6 months (Table 3). The prevalence of inappropriate malaria care was lower in participants that received healthcare in Abim general hospital compared to those that received healthcare from Kaabong general hospital (aPR = 0.4; 95% CI 0.3–0.5, p < 0.001). The prevalence of inappropriate malaria care was higher if patients visited the health facilities when there was a stock out of recommended anti-malarials (aPR = 2.1; 95% CI 1.8–2.4, P < 0.001). Participants were more likely to receive inappropriate malaria care when they were managed by a clinician who had not received support supervision in the last 6 months than when they were managed by a clinician who had received support supervision (aPR = 0.5; 95% CI 0.2–0.9, p value = 0.030). Finally, the prevalence of inappropriate malaria care was higher in participants managed by clinical officers than other cadres, Table 3.

**Table 3:** Factors associated with inappropriate malaria care among 857 children under-5 years in Kaabong and Abim General Hospitals

Characteristic		Inappropriate care n (%)	Crude PR (95% CI)	P-value	Adjusted PR (95% CI)	P-value
Study site	Kaabong	249(58.2)	1		1	
	Abim	72(16.8)	0.3 (0.2–0.4)	**< 0.001**	0.4 (0.3–0.5)	**< 0.001**
Child’s age	≥ 6 months	292(36.9)	1		1	
	< 6 months	29(43.9)	1.1 (0.7–1.9)	0.633*	1.1 (0.9–1.3)	0.172
History of travel prior to symptom onset	No	299(38.5)				
	Yes	22(27.5)	0.9 (0.6–1.2)	0.360		
Time to care for fever	> 1 day	258(37.8)	1		1	
	≤ 1 day	63(36.2)	0.9 (0.8–1.0)	**0.104**	0.9 (0.7–1.0)	0.062
Prior treatment with anti-malarial	No	230(36.9)	1			
	Yes	91(39.1)	1.2 (0.9–1.5)	0.232		
Employment status	Unemployed	152(39.2)	1		1	
	Informal	163(38.0)	1.1 (0.6–2.2)	0.735	0.8 (0.6–1.1)	0.220
	Formal	6(15.0)	0.8 (0.7–0.9)	**< 0.001**	0.8 (0.7–0.9)	0.072
Education status	None/Primary	277(39.7)	1			
	Secondary/tertiary	44(27.7)	1.0 (0.8–1.3)	0.847		
Recommended anti-malarials available	Yes	202(27.8)	1		1	
	No	119(90.8)	2.4 (1.9–2.9)	**< 0.001**	2.1 (1.8–2.4)	**< 0.001**
Health worker cadre	Clinical officer	268(36.3)	1		1	
	Doctor	46(43.4)	1.0 (0.9–1.2)	0.732	0.8 (0.7–0.9)	**< 0.001**
	Nursing officer	7(53.9)	1.8 (1.0–3.4)	**0.057**	0.7 (0.6–0.8)	< 0.001
Managed by a clinician supervised in last 6 months	No	316(39.8)	1		1	
	Yes	5(8.1)	0.3 (0.2–0.5)	**< 0.001**	0.5 (0.2–0.9)	**0.030**
Managed by a clinician with malaria training in last 1 year	No	244 (49.8)	1		1	
	Yes	77(21.0)	0.7 (0.4–1.3)	0.293*	0.9 (0.8–1.3)	0.974

## Discussion

This cross-sectional study evaluated the malaria diagnostic and treatment practices, as well as the factors associated with inappropriate practices for children under 5 years presenting with fever in the two public hospitals in the Karamoja sub-region. The study found that there is a high prevalence of inappropriate malaria care (diagnosis and/or treatment); with approximately one in every three children under five receiving inappropriate malaria care. Factors significantly associated with inappropriate malaria care included the health facility where the patient sought care, stock out of recommended anti-malarials, the cadre of the healthcare worker who provided the malaria care, and whether the clinician had received any support supervision in the last 6 months or not. These findings highlight the high prevalence of inappropriate malaria care in children under the age of five in the Karamoja sub-region, which falls short of the national target of 90% of all suspected malaria cases receiving appropriate malaria care [6, 9]. Given that appropriate malaria care is one of the key elements in lowering malaria-related mortality, this high prevalence of inappropriate case management may be one of the numerous causes of the region’s high malaria-related mortality in children under the age of five.

These results indicate that at least one in every three children under 5 years of age that present with fever at the two of the main public health facilities in the Karamoja sub-region receive inappropriate malaria care by either missing a malaria diagnostic test, or receiving inappropriate malaria treatment. Malaria diagnosis is the first step in case management without which, case management may be compromised, necessitating presumptive malaria treatment. Presumptive case management contributes to: (1) anti-malarial drug wastage due to over-diagnosis of malaria, which is a major contributor to increased stock out periods during which true malaria cases cannot receive the life-saving medicines [3]; (2) increases the risk of exposing patients to unnecessary side effects and treatment costs; (3) increases the risk of development of resistance to the available anti-malarials; and (4) may deny the opportunity to investigate alternative causes of the patient’s disease, which may result poor clinical outcomes when patients are incorrectly managed [13, 14]. In this study, when a test was not requested, almost all the time participants received presumptive treatment.

The basic determinant of effective malaria case management is the availability of “test and treat” health commodities and from this study, it was observed that malaria diagnostic tests were readily available at the participating health facilities. Although artemether/lumefantrine tablets, the recommended first-line drug for uncomplicated malaria, were available at both study health facilities throughout the study, IV artesunate ampoules; the recommended treatment for severe cases, were stocked out in Kaabong for almost half of the study period. It is, therefore, not surprising that the largest contributor to inappropriate malaria care was prescription of anti-malarials other than the recommended first-line therapy, with Kaabong hospital contributing highest to this percentage. Despite the availability of artemether/lumefantrine, dihydroartemisinin/piperaquine tablets (Duocotexin®) (the rescue treatment in cases of treatment failure with the recommended first-line) and quinine tablets/ syrup were often used as first line treatment for uncomplicated malaria in this setting. Similarly, to what has been previously observed, quinine injection was the most common used alternative anti-malarial for severe malaria. Quinine has been documented to be associated with poor adherence as a result of the complexities of its dosing and toxicity profile [15–19]. Given this evidence, quinine should not be used to treat malaria when artemisinin-based combinations are available, emphasizing the need to reduce ACT wastage.

It is important to note that even with this under performance, all children under five who had a positive malaria test in this study received an anti-malarial, and less than 20% of test negative children received an anti-malarial; a proportion less than what has been previously observed in other studies in Uganda and the East African region [20−23]. This observation suggests that in this study setting, there is more confidence in reliability of malaria diagnostic tests contrary to what has been previously reported as reason for health workers prescribing an anti-malarial to patients with cardinal malaria signs but having negative test results [24, 25]. Despite this observation, there is a need to improve adherence to test results in order to facilitate effective management of non-malaria febrile illnesses that, if not managed properly, can result in death [3].

Beyond stock out of recommended anti-malarials, the cadre of the clinician and support supervision to healthcare workers were factors significantly associated with inappropriate malaria care in this study. Controversy exists on the impact of support supervision on adherence to treatment guidelines. As observed in this study, other studies have reported support supervision to positively impact adherence to treatment guidelines [26, 27] and have attributed this to the fact that support supervision helps improve and maintain performance, therefore, necessitating its continuous implementation in many endemic countries. On the other hand, other studies including one carried out in Uganda have reported no association between support supervision and appropriate patient care [12]. However, the lack of association observed in these studies could be that in most cases, support supervision is inadequate or unfocused resulting in no impact. For support supervision to be effective, it must be systematic (with inputs; the right supervisors with the necessary tools and resources for planning and implementation) and have an active feedback and follow-up channel in order to effect change and enhance compliance with guidelines [28].

Due to a human resource shortage, clinical officers constitute the backbone of Uganda’s health-care system at the general hospital level in rural locations like Karamoja.

This translates to more children being managed by clinical officers than by any other cadres because doctors are typically hospital heads and are more likely to be supervised and trained which improves their adherence to guidelines, whereas nurses are less likely to be involved in diagnosis and treatment at this level. This might explain why, when compared to clinical officers, doctors and nurses were less likely to inappropriately manage children under five, which is consistent with previous studies [29].

Training should precede policy implementation if it is to achieve the intended objectives, as proven by earlier studies that demonstrated an association between training and policy adherence [26, 30–32]. That is what was hypothesized, as training has been shown to enhance understanding and adherence. However, this study no significant association between training and malaria care, which is consistent with the findings of another Ugandan study [12]. This observed lack of association may be due to inadequacy in training quality and duration, though this can be contested by results from different studies conducted in Dar-es-Salaam and Kenya which reported improvement in adherence after a half to one day’s training [33, 34], whereas poor adherence was reported in a Burkina Faso study after a three-day training [35].

Age is also an important factor in the management of malaria in children under the age of five, as fewer infections are expected in children younger than 6 months because they are protected against malaria by maternal antibodies, so a malaria diagnosis is the last option after all other fever causes have been investigated in this age group. As a result, it was hypothesized that children older than 6 months were more likely to be inappropriately managed for malaria compared to their younger peers. Surprisingly, contrary to the findings of another Ugandan study [36], this study found no association between age and inappropriate malaria care. This disparity might be explained by the fact that the number of children under the age of 6 months in the current study was insufficient to detect a significant difference.

## Study limitations

First, quantitative study methods used in the current study did not allow us to explore how and why questions of the prevalence of inappropriate malaria care and the mechanisms underlying the association with the identified factors. A qualitative research study to better examine and understand these phenomena is recommended to better understand this.

Second is the possibility of Hawthorne effect bias, which might have resulted in an underestimation of the prevalence of inappropriate management with more health workers adhering to treatment guidelines during the research period than they would ordinarily. However, it was mitigated by conducting interviews with health workers at the end of the study period. Third, given that malaria case management was assessed based on a single visit, it was not possible to estimate the treatment outcomes based on the level of care received and thus are unable to confirm if the observed high levels of inappropriate care are the reasons that there is a high malaria related mortality in the region.

Finally, the study was conducted at general hospitals, which may present different challenges (in terms of case management commodities stocking, health professional staffing and management) than lower levels of the public health system which implies that these study findings may apply only to the level of care of general or district hospitals.

## Conclusion

Inappropriate malaria diagnosis and/or treatment is common, with one in every three children under five receiving inappropriate malaria care, and is mainly driven by health system factors such as availability of recommended malaria commodities and support supervision of the attending healthcare workers. The greatest contributor was lack of recommended anti-malarials at the health facility and, therefore, strategies to improve malaria management commodity supply such as the sustainable implementation and training on stock monitoring systems in health facilities should be prioritized.

To help solve the problem of frequent stock outs and inappropriate malaria care, capacity of health facilities to ensure that inputs for appropriate malaria care are available at the same time is imperative. These include the recommended commodities for malaria diagnosis and treatment, fever management, trained healthcare workers and supported pharmacy professionals, treatment guidelines, job aids, treatment algorithms, chart reminders, and stock management procedures and processes.

Trainings alone are insufficient to influence change in the adoption of new policies, and should be combined with focused support supervision, which has been shown to significantly affect case management.

## Data Availability

Data set and study materials used during the study are available from corresponding author on reasonable request.

## Abbreviations

ACT: Artemisinin-based combination therapy
AL: Artemether-lumefantrine
DHIS 2: District Health Information Software
DHO: District Health Officer
iCCM: Integrated Community Case Management
IPD: In-Patient Department
IRS: Indoor residual spraying
LLINs: Long-lasting insecticidal nets
MCM: Malaria case management
MOH: Ministry of Health
OPD: Out-Patient Department
PI: Principal investigator
RDT: Rapid Diagnostic Test
SOMREC: School of Medicine Research and Ethics Committee
WHO: World Health Organization
